# Broadband light management in hydrogel glass for energy efficient windows

**DOI:** 10.1007/s12200-022-00033-4

**Published:** 2022-08-05

**Authors:** Jia Fu, Chunzao Feng, Yutian Liao, Mingran Mao, Huidong Liu, Kang Liu

**Affiliations:** 1 MOE Key Laboratory of Hydraulic Machinery Transients, School of Power and Mechanical Engineering, Wuhan University, Wuhan, 430072 China; 2 Wuhan National Laboratory for Optoelectronics, Huazhong University of Science and Technology, Wuhan, 430074 China

**Keywords:** Hydrogel, Light management, Windows, Energy saving, Broadband

## Abstract

Windows are critically important components in building envelopes that have a significant effect on the integral energy budget. For energy saving, here we propose a novel design of hydrogel-glass which consists of a layer of hydrogel and a layer of normal glass. Compared with traditional glass, the hydrogel-glass possesses a higher level of visible light transmission, stronger near-infrared light blocking, and higher mid-infrared thermal emittance. With these properties, hydrogel-glass based windows can enhance indoor illumination and reduce the temperature, reducing energy use for both lighting and cooling. Energy savings ranging from 2.37 to 10.45 MJ/m^2^ per year can be achieved for typical school buildings located in different cities around the world according to our simulations. With broadband light management covering the visible and thermal infrared regions of the spectrum, hydrogel-glass shows great potential for application in energy-saving windows.

**Graphical Abstract:**

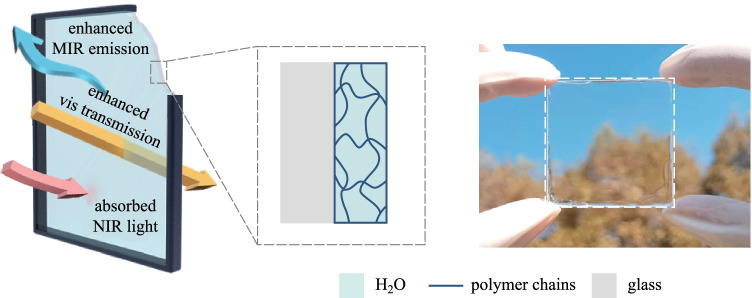

**Supplementary Information:**

The online version contains supplementary material available at 10.1007/s12200-022-00033-4.

## Introduction

Energy use in buildings contributes to over 40% of the world’s total energy consumption, of which lighting and space cooling make up a significant proportion [1, 2]. Traditional glass windows are the least-energy-efficient components in buildings [3, 4]. In the summer, the near-infrared (NIR) sunlight transmitted through windows produces into undesired heating [4–6]. In turn, the high reflection of mid-infrared (MIR) limits heat rejection from the building. This “greenhouse effect” aggravates cooling energy consumption [7]. In the past decades, a lot of efforts have been devoted to manipulation of the optical properties of windows, to save energy in buildings.

Radiative cooling that passively dumps heat into the cold outer space via MIR electromagnetic waves through the atmosphere window is an attractive strategy for building cooling [8–11]. Visible transparent coatings with high emissivity in MIR have been proposed to enhance heat dissipation by way of windows [12–15]. However, they do not block the NIR sunlight. Low-E glass can reflect the NIR sunlight to mitigate internal building heating, but the low emissivity of MIR induces heat accumulation and low visible transmittance leads to higher lighting demand [16, 17]. Electrochromic windows, which can switch the NIR transmittance with an applied electric field, have also been developed to improve building energy efficiency [18]. However, the narrow working spectrum of these approaches distances the windows from optimized efficiency, which requires the manipulation of electromagnetic spectrum covering visible, NIR and MIR [19, 20]. Novel windows with broadband light management capability are still highly desirable in order to improve energy-efficiency.

Hydrogels are formed through the cross-linking of hydrophilic polymer chains within an aqueous environment. The water-rich and transparent nature makes them a potential choice for window engineering. Based on its thermo-responsive property, Zhou and Li et al. used hydrogels to fabricate thermochromic hydrogel glass with visible transparency varying with environmental temperature [21, 22]. However, these works focus only on the visible manipulation and temperature responsiveness. The inherent property of hydrogels in near-infrared and mid-infrared, and the potential benefit of low refraction index, are not investigated.

**Fig. 1:**
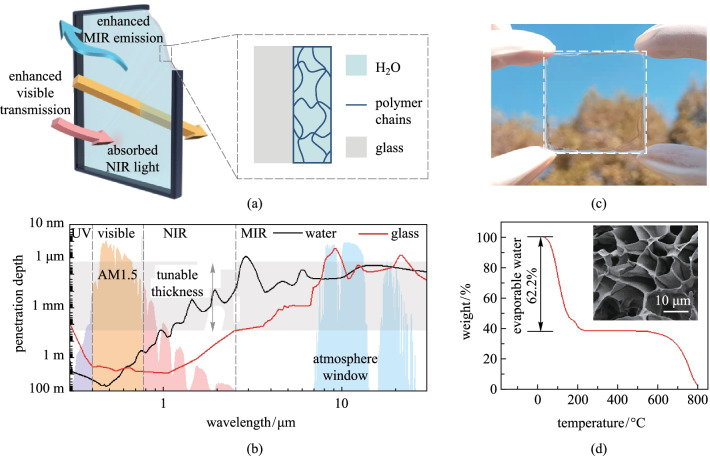
Structure design and working principle of the hydrogel-glass. **a** Schematical structure of the hydrogel-glass with the functions of enhanced visible transmission, absorbed NIR light and enhanced MIR emission. **b** Calculated photon penetration depths for liquid water and traditional silica glass. **c** Photograph of the fabricated hydrogel-glass. **d** Thermogravimetric analysis of the hydrogel. Inset is the SEM image of the freeze-dried hydrogel

In this work, we propose a hydrogel-glass (Fig. 1a) with broadband light management capability covering the visible, NIR and MIR spectrum and we investigate its performance in energy-saving windows. First, we introduced the fabrication of the hydrogel glass and the basic optical properties in the visible, NIR and MIR regions of the electromagnetic spectrum. Then, the optical and thermal performances of the hydrogel glass were demonstrated in a solar cell and a hand-made simulated house. Lastly, the energy saving potential of the application of hydrogel glass in windows in a school building was estimated via the simulation with EnergyPlus software.

## Methods

### Preparation of the hydrogel-glass

The hydrogel-glass was prepared by directly photo-polymerizing the hydrogel precursor solution on the glass surface. First, the glass surface (40 mm × 40 mm × 2 mm) was activated by air plasma (10.5 W) for 10 min, during which hydroxyl groups were bonded onto the glass surface. Then, the substrate was immersed into mixed solution containing 3 mL 3-(trimethoxysilyl) propyl methacrylate, 20 µL acetic acid and 150 mL deionized water for 12 h. During the immersion, the silane coupling agents bonded with the hydroxyl groups, forming a layer of silanol groups on the glass surface [23]. Subsequently, the glass was put into a mold thicker than the glass and the precursor solution, containing 2 mol/L acrylamide, 0.001 mol/L N, N′-methylenebis(acrylamide) and 0.002 mol/L 2-hydroxy-4′-(2-hydroxyethoxy)-2-methylpropiophenone, was poured into the mold. Then the mold was clamped by two pieces of glass substrates and irradiated under ultraviolet light (365 nm, ca. 4 mW/cm^2^) for 8 h with nitrogen protection, obtaining the polyacrylamide (PAAm) hydrogel bonded glass. Here, the thickness of hydrogel was determined by the thickness of the mold. Finally, the hydrogel-glass was soaked in aqueous LiBr solution (8 mol/L) until it was swollen completely, obtaining the hydrogel-glass. The LiBr was introduced into the hydrogel to prevent dehydration.

### Characterizations

The morphologies of the dry hydrogel were characterized by a scanning electron microscope (TESCAN, MIRA3). Thermogravimetric analyzer (TGA 4000, PerkinElmer) was used to measure the water content in the hydrogel. The tests were conducted in the temperature range from 30 to 800 °C with a scanning rate of 15 °C/min. The bonding strength between the hydrogel layer and the glass layer was measured by a universal mechanical test machine (CMT6350, SANS) at a stretching rate of 15 mm/min. Mass variations of hydrogels were measured by an electronic balance (ML-T, Mettler Toledo). Spectral reflectance (*R*) and transmittance (*T*) in 0.3–2.5 μm were measured using a UV–Vis-NIR spectrophotometer (Lambda 1050, Perkin Elmer) equipped with an integrating sphere (Labspher8). Spectral reflectance (*R*) in the range 2.5–25 μm was measured by a Fourier transform infrared spectrometer (FTIR, INVENIO S, Bruker) with a gold integrating sphere (A562). The spectral absorptance (*A*) can be calculated by *A* = 1 − *R* − *T* in the solar spectrum (AM1.5), and by *A* = 1 − *R* in MIR spectrum (*T* = 0).

### Performance measurements

In the laboratory environment, the photocurrent density–voltage curves of the solar cells with different thick hydrogel layers were characterized by a Keithley 2400 source meter. Temperature of the cell was measured using a thermocouple (TT-K-30, Omega Company) fixed at the bottom of the cell and recorded by a data logger (TC-08, Pico Technology). The simulated solar light was provided by a solar simulator (SS-100A, Class AAA, Sanyou Corporation). The solar intensity was measured by an optical power meter (CEL-NP2000-2).

To measure the radiation cooling performance, solar cells with normal glass and hydrogel-glass were placed in a chamber, made of the polystyrene foam to minimize the influence of environment effects. Temperatures of the solar cells were measured by two thermocouples fixed in the center of the bottom side. Another thermocouple was put in the chamber to measure the ambient temperature. The relative humidity of the environment was monitored using a thermo-hygrometer (CENTER 310).

To demonstrate the visible and NIR light management of the hydrogel glass, two model houses with the same size of 20 cm × 20 cm × 20 cm were used for outdoor experiments. The walls and floor of the houses were made of an outer layer of 2-cm-thick glued wood and an inner layer of 3-cm-thick polystyrene foam to minimize heat leakage. One of the houses was covered by a hydrogel-glass window (3-mm-thick hydrogel on a 6-mm-thick glass substrate). The other house was covered with a 6-mm-thick glass for comparison. In each house, the indoor illuminance and temperature were measured by a split type lux meter (DELIXI DLY-1801) with a silicon detector (working wavelength range in 400−900 nm) and a thermocouple, respectively. The lux meter was placed on the center of the floor of each house, and the thermocouple was in the air about 3 cm above the floor. The temperature of the hydrogel glass was measured with a thermocouple fixed between hydrogel and glass layers. A thermocouple was fixed on the top of the normal glass via glue to measure the temperature. The outdoor solar intensity was recorded by the optical power meter (CEL-NP2000-2).

### Simulations

The energy-saving of the hydrogel-glass was simulated by the EnergyPlus (version 9.5.1) software. A typical primary school building model from the EnergyPlus database was employed. The total building area was 6871 m^2^, and the building exterior surface included walls (2473.6 m^2^), roofs (3518.26 m^2^), vertical windows (865.76 m^2^), and horizontal skylights (13.38 m^2^). The total window opening area was 879.14 m^2^.

The optical and thermal properties of windows used in the simulations are listed in Additional file 1: Table S1. Normal glass (CLEAR 6MM) was selected from the EnergyPlus database. The parameters of transparent NIR shielding (TNS) glass was obtained from the reference [24]. The optical and thermal parameters of hydrogel-glass were calculated from the measured data. (Details about the calculation can be found in Additional file 1: Note 1 and Note 2). The weather data of each city were obtained from the EnergyPlus database website [25].

In the simulation model, extra heating or cooling was needed to balance the heat transfer between the internal building and the environment, so as to maintain a fixed indoor temperature. The extra power can be calculated:

1 $${P}_{\mathrm{gen}}^{ }+{P}_{\mathrm{sun}}={P}_{\mathrm{conv}}+{P}_{\mathrm{cond}}+{P}_{\mathrm{rad}}+{P}_{\mathrm{extra}},$$

where *P*_gen_ is the heat generated by the internal loads, *P*_sun_ is the absorbed solar flux by the school building (roofs, walls and windows). The right side of the equation represents the heat exchange between the building and the environment. Here, *P*_conv_, *P*_cond_, and *P*_rad_ are the net convective, conductive, and radiative heat fluxes, respectively. *P*_extra_ is the extra power to maintain the room temperature at a determined value.

The lighting energy consumption was calculated from the EnergyPlus daylighting model using the SplitFlux method [26]. Indoor brightness is maintained at 300 Lux by both the natural and artificial lighting.

## Results and discussion

The hydrogel-glass consisted of a layer of hydrogel and a layer of normal glass (Fig. 1a). The hydrogel layer was composed of small amount of cross-linked polymer and large amount of liquid water. Hence, it possessed similar optical properties to those of water [27, 28]. As shown in Fig. 1b, traditional glasses have large photon penetration depth in the whole solar spectrum without the capability to filter out NIR from sunlight (Additional file 1: Note 3). As a comparison, the photon penetration depth of water is greater than 1 m for most of the visible spectrum, and rapidly decreases to several millimeters for most of the NIR spectrum. Thus, the hydrogel can let through incoming visible light and block incoming NIR. Moreover, according to the figure, the transmittance of the hydrogel can be tuned by its thickness to obtain a desirable transmittance spectrum. While in the wavelengths corresponding to infrared transparency window of atmosphere, both water and glass have low penetration depth, which means they are nontransparent even with the thickness of 100 μm. Here the penetration depth of the glass has the smallest value of about 0.3 μm around the wavelength of ~ 9 μm, which is caused by the Si–O–Si asymmetric stretching vibrations [29, 30]. The strong lattice variation in the glass also means it has a high refraction index, high surface reflection and low thermal emittance [12]. However, no strong lattice or molecular vibration exist in the water or hydrogel [30]. Therefore, it is possible that the hydrogel-glass can enhance the MIR emission, block most NIR sunlight, and keep high transmission of visible light (Fig. 1c).

Thermogravimetric measurement showed that vaporizable water in the hydrogel accounts for 62.2% of the total weight, an equivalent volume fraction of ~ 95% in the hydrogel (Fig. 1d). SEM image of the freeze-dried hydrogel clearly revealed the network of solid polymer, which helped to confine liquid water inside the hydrogel (Fig. 1d). In addition, the LiBr salt inside reduced the vapor pressure of water inside to balance with the ambient humidity [31, 32]. Hence, the hydrogel retained its weight and structure stability under varying environmental conditions, as shown in Additional file 1: Fig. S1. The hydrogel layer was also firmly bonded with the glass substrate, as illustrated by the stress–strain curve in Additional file 1: Fig. S2.

**Fig. 2:**
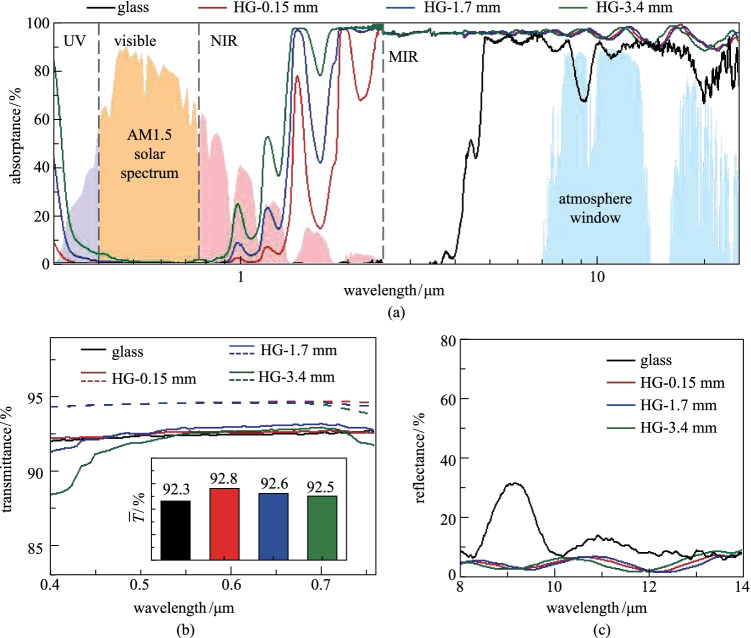
Spectral characteristics of the hydrogel-glass with different-thickness hydrogel layers compared with traditional glass. **a** Measured spectral absorptance in the wavelength range from 0.3 to 25 μm. Here, HG in the figure is abbreviation of hydrogel-glass. **b** Spectral transmittance in visible light. The solid and dashed lines represent the measured and calculated results, respectively. The inset chart shows the average transmittance ($\overline{T }$). **c** Measured spectral reflectance in the wavelengths of infrared transparency window of atmosphere

We further characterized the spectral absorptance of the hydrogel-glass in the wavelength range from 0.3 to 25 μm (Additional file 1: Fig. S3). The absorptance of traditional glass was also presented for comparison. As shown in Fig. 2a, the hydrogel-glass had extremely low absorptance approaching zero in the visible light, which is very similar to traditional glass. In the NIR spectrum, the hydrogel-glass had multiple absorption peaks originating from the O–H stretching vibrations [33]. With increase of the thickness of hydrogel layer, the hydrogel-glass presented higher absorptance, and a hydrogel layer with several millimeter thick could block most NIR sunlight. In the atmosphere window spectrum, the hydrogel-glass had higher absorptance than the glass, which means hydrogel-glass had higher thermal emittance. To further confirm the measured results, we calculated the theoretical spectral absorptance of hydrogel layer on a glass substrate based on the model presented in Additional file 1: Fig. S4. Detailed calculation can be found in Additional file 1: Note 1. As shown in Additional file 1: Fig. S3, the theoretical spectral absorptances were close to the measured results.

We also measured the transmittance of the hydrogel-glass in the visible spectrum from 0.38 to 0.76 μm. As shown in Fig. 2b, the hydrogel-glass had slightly higher transmittance than the glass substrate in most of the visible spectrum. The average transmittances ($\overline{T }$) of hydrogel-glass in visible spectrum were calculated based on Additional file 1: Eq. (S6). As shown in the inset chart of Fig. 2b, $\overline{T }$ of the glass substrate was 92.3%. While the average transmittance of the hydrogel-glass was 92.8% with a 0.15-mm-thick hydrogel layer. Two reasons are responsible for the enhanced visible transmittance: firstly, water has a negligible extinction coefficient in the visible range; secondly, the refractive index of water (1.33) is lower than that of glass (1.5), reducing the surface reflection of incident light. When further increasing the hydrogel thickness to 1.7 and 3.4 mm, $\overline{T }$ decreased to 92.6% and 92.5%, respectively. Figure 2c presents the spectral reflectance of the hydrogel-glass and normal glass in the wavelengths of infrared transparency window of atmosphere. The glass exhibited a reflection band at about 9 μm due to the strong refraction caused by the Si–O–Si asymmetric stretching vibrations [29], and this led to a low thermal emittance of ~ 0.84. In contrast, the surface reflectance of the hydrogel-glass was lower than 5% in the wavelengths of infrared transparency window of atmosphere. By integrating the spectral absorption over the wavelength with respect to black body radiation (Additional file 1: Eq. (S7)), we obtained a high thermal emittance of ~ 96%, which is higher than that of other transparent radiative cooling windows [12, 34]. The results of transmittance and reflectance of hydrogel-glass in the full region of the electromagnetic spectrum can be found in Additional file 1: Fig. S3b and 3c.

**Fig. 3:**
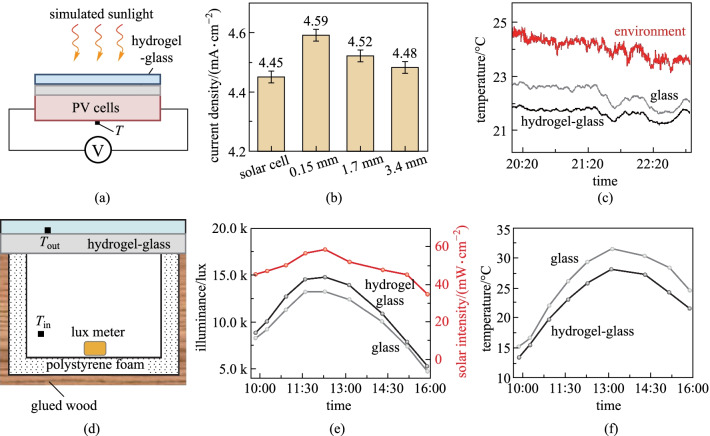
Demonstrations of the broadband light management performance of the hydrogel-glass. **a** Schematic of the solar cell with hydrogel-glass. **b** Current density of the solar cell with different thickness of hydrogel layers. **c** Temperatures of the solar cell, solar cell with hydrogel-glass and environment from 20:00 to 23:00 at outdoor (May 21, 2021 in Wuhan, China). The relative humidity was ~ 70%. **d** Schematic of a house with a piece of hydrogel-glass as the window. Here, the 3-mm-thick hydrogel was used due to its high NIR absorption. **e** Illuminance inside the house with the hydrogel-glass and common glass in a sunny day (Jan. 1st, 2022). **f** Indoor temperatures inside the houses with hydrogel-glass and common glass

To demonstrate the broadband light modulation capability, we simply put the hydrogel layer on a commercial glass encapsulated Si solar cell, as shown in Fig. 3a and Additional file 1: Fig. S5a. Under solar irradiance of 1 kW/m^2^ from the sunlight simulator, the solar cells with hydrogel-glass presented higher photocurrent densities and efficiencies than those for the cells encapsulated with normal glasses, as shown in Fig. 3b and Additional file 1: Fig. S5b. The photocurrent density increased from 4.48 to 4.59 mA/cm^2^ with the thickness of hydrogel decreasing from 3.4 to 0.15 mm. The temperature of the solar cells decreased with the decreasing thickness due to the higher surface reflectance in the solar spectrum (Additional file 1: Figs. S5c and d), indicating that smaller thickness is more suitable for enhancing photovoltaic efficiency. Here it is important to note that, the hydrogel-glass enhances the thermal emittance in the wavelengths of infrared transparency window of atmosphere, and can also induce strong radiative cooling and possibly reduce the temperature of solar cells in the working mode. As shown in Fig. 3c and Additional file 1: Fig. S6, the temperature of a hydrogel glass in an outdoor environment is obviously lower than the temperature of traditional glass and ambient temperature, indicating strong radiative cooling. Thus, it is predicted that the efficiency of the solar cell with hydrogel glass can be even higher through heat dissipation to outer space [35, 36].

To further demonstrate the potential of the hydrogel-glass for windows in buildings, we set the hydrogel-glass on a small house model with a size of 20 cm × 20 cm × 20 cm, and measured the illuminance and temperature at different spots (Fig. 3d and Additional file 1: Fig. S7a). Figure 3e shows that the indoor illuminance of the house with hydrogel-glass is slightly higher than that in the house with normal glass in a sunny day. The enhanced illuminance helps to reduce the electricity consumption from lighting. More importantly, the indoor temperature (*T*_in_) of the house with the hydrogel-glass is always lower than that of the house with normal glass as shown in Fig. 3f. The largest temperature reduction reaches 3.5 °C at noon with the highest solar intensity of 58.7 mW/cm^2^. Hence, the hydrogel-glass can reduce the cooling power consumption of a building in the summer. The reduction of the indoor temperatures is mainly caused by the absorption of NIR sunlight in the hydrogel-glass; the temperatures of the hydrogel-glass (*T*_out_) were always higher than those of the normal glass, as presented in Additional file 1: Fig. S7b.

**Fig. 4:**
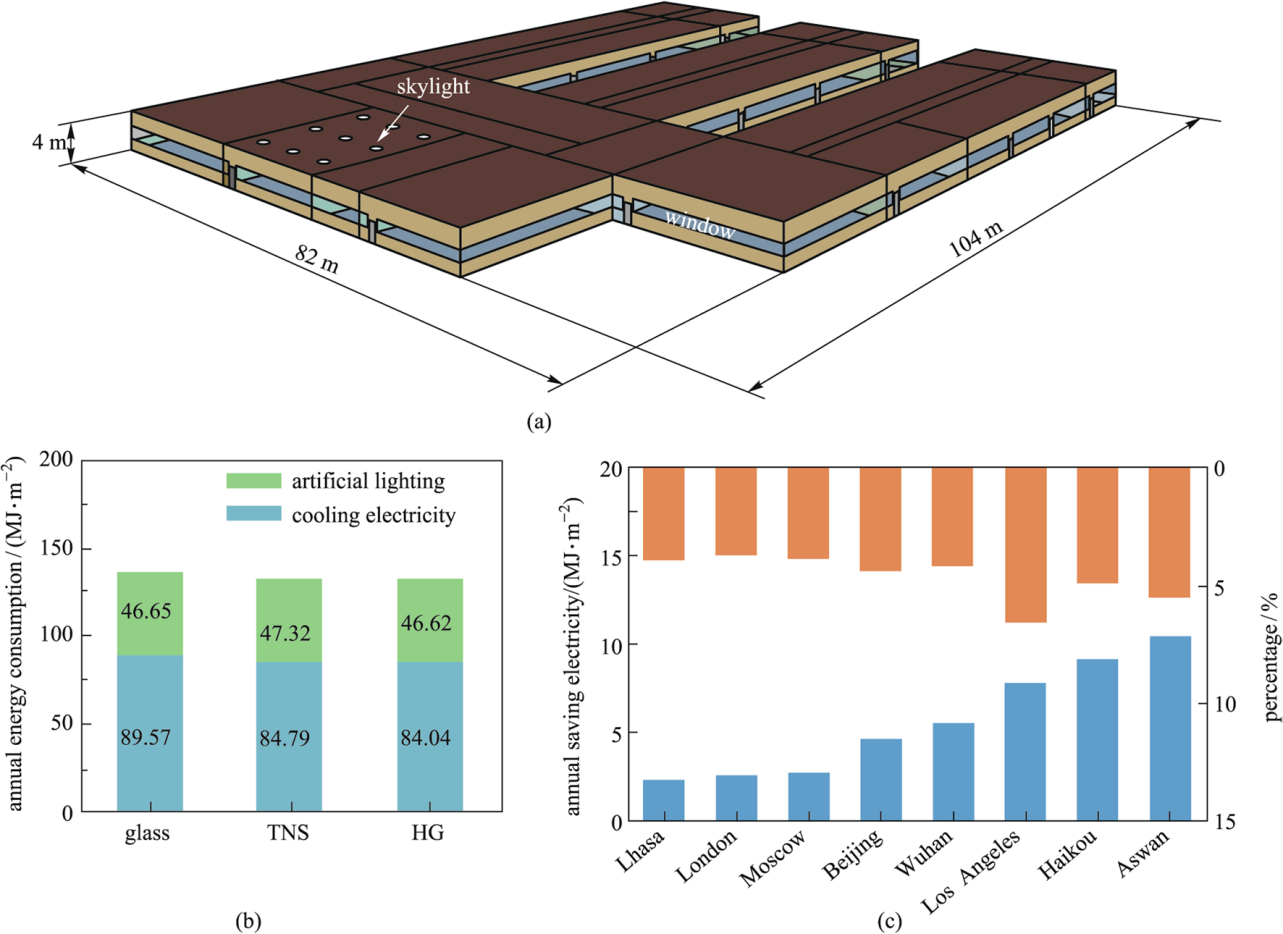
Energy saving evaluation of the hydrogel-glass as windows in buildings. **a** Schematic of a school building. **b** Annual energy consumption of the school building based on weather data for Wuhan, China (30.62° N, 114.13° E). Three windows of normal glass, TNS, and hydrogel-glass were used in the simulations. **c** Annual energy savings and the corresponding percentage of building models with hydrogel-glass in eight cities around the world.

To evaluate the energy-saving potential of the hydrogel-glass in buildings, we calculated the energy consumption of a typical building, using EnergyPlus, considering the daytime lighting and indoor temperature regulation. As shown in Fig. 4a, the building model was a school with two types of windows including the vertical windows in walls and horizontal skylights in roofs. The windows accounted for 13% of the external surface area. The energy consumptions of normal glass and TNS glass were also calculated for comparison [24]. Detailed optical and thermal parameters of the three types of glass are summarized in Additional file 1: Table S1.

We first evaluated the annual energy consumption per building area for lighting and cooling of the building model, assuming its location to be in Wuhan, which has a subtropical monsoon climate (Additional file 1: Table S2). As shown in Fig. 4b, the normal glass was calculated to have annual cooling and lighting energy consumption of 89.57 and 46.65 MJ/m^2^, respectively. The use of TNS glass reduced the cooling energy to 84.79 MJ/m^2^, but increased the lighting energy demand to 47.32 MJ/m^2^. In contrast, the use of hydrogel-glass reduced both the cooling and lighting energy to 84.04 and 46.62 MJ/m^2^, respectively. The annual energy saving was calculated to be up to 5.56 MJ/m^2^ as compared with that for normal glass (Fig. 4c). The annual electricity savings in eight representative cities (Additional file 1: Table S2) with different climatic conditions were also calculated [25]. As shown in Fig. 4c, the calculated energy savings were ranged from 2.37 to 10.45 MJ/m^2^, accounting for ~ 3% to ~ 8% of annual energy consumption. Among them, the Aswan city in the dry and hot tropical desert climate has the highest potential for energy saving up to 10.45 MJ/m^2^. In cities with longer annual lighting time such as Moscow, the hydrogel-glass was evaluated to have the highest annual lighting energy saving of 0.05 MJ/m^2^, as shown in Additional file 1: Table S3. These results suggest the great potential of hydrogel-glass for energy saving in different climate conditions.

## Conclusions

In summary, we have developed a novel hydrogel-glass by coating environmentally stable hydrogel onto traditional glass. With the low refraction hydrogel layer, the hydrogel-glass increases the visible transparency of windows and reduces the electricity consumption for building illumination. In addition, it blocks most of the NIR sunlight and rejects the heat into outer space via enhanced mid-infrared emittance, and thus reduces the cooling power demand. Its absorption, transmission and reflection properties at different wavelengths endow the hydrogel-glass with capability of simultaneous solar and thermal management. Based on simulations, the hydrogel-glass can achieve energy savings ranging from 2.37 to 10.45 MJ/m^2^ per year for building models located at different cities around the world, providing a possible approach for next-generation energy-efficient windows.

## Supplementary Information

**Additional file 1.** **Note 1.** Theoretical calculations of the reflectance, transmittance and absorptance of the hydrogel-glass. **Note 2.** Thermal conductivity of the hydrogel-glass. **Note 3.** Photon penetration depth of water and glass. **Figure S1.** Mass variation of the hydrogel exposed in ambient environment. The size of the samples is 1cm × 2 cm × 2 mm. The ambient temperature is about 26 °C, and the relative humidity is about 50%. Inset shows the hydrogel after storage in environment for two years and the newly prepared hydrogel. **Figure S2.** Stress-strain curve of the hydrogel sandwich between two glass plates. Inset is the photograph during the test. **Figure S3.** Optical spectra of the glass and hydrogel-glasses. (**a**) Theoretical and measured spectral absorptance in region of 0.3-25 μm. Here, it is noted that the liquid water has no absorption in the UV spectrum according to the calculation. The measured absorption of the hydrogel is caused by the polymer networks in the hydrogel. The solid and dashed line represent the measured and calculated results, respectively. (**b**-**c**) Measured spectral transmittance (**b**) and reflectance (**c**) in the range of 0.3-2.5 μm. **Figure S4.** Schematic of the calculations of the transmittance and reflectance of the hydrogelglass. **Figure S5.** Light management of solar cells with hydrogel-glass with different thick hydrogel layers. (**a**) Photograph of the solar cells. The ccale bar is 4 cm. (**b**-**c**) Photovoltaic efficiencies (**b**) and working temperatures (**c**) of the solar cells.(**d**) Measured spectral reflectance of the solar cells in 0.3-2.5 μm. **Figure S6.** Experiment setup to test the radiative cooling performance of hydrogel-glass. (**a**) Schematic of the setup. (**b**) Photograph of the experiment setup. **Figure S7.** Performance demonstration of the hydrogel-glass in a house model. (**a**) Photograph on experiment setup. (**b**) Temperatures of the hydrogel-glass, normal glass and environment. **Table S1** Optical and thermal properties of three windows used in the simulations. **Table S2** Climatic classification and geographical location of eight cities. **Table S3** Annual electricity consumption and energy saving potential in the eight cities.
