# Supplementary material for: Broadband light management in hydrogel glass for energy efficient windows
Source: Front Optoelectron. 2022 Aug 5;15(1):33. doi: 10.1007/s12200-022-00033-4 (PMC9756254; doi:10.1007/s12200-022-00033-4)
Supplement: Supplementary file 1 — Additional file 1. Note 1. Theoretical calculations of the reflectance, transmittance and absorptance of the hydrogel-glass. Note 2. Thermal conductivity of the hydrogel-glass. Note 3. Photon penetration depth of water and glass. Figure S1. Mass variation of the hydrogel exposed in ambient environment. The size of the samples is 1cm × 2 cm × 2 mm. The ambient temperature is about 26 °C, and the relative humidity is about 50%. Inset shows the hydrogel after storage in environment for two years and the newly prepared hydrogel. Figure S2. Stress-strain curve of the hydrogel sandwich between two glass plates. Inset is the photograph during the test. Figure S3. Optical spectra of the glass and hydrogel-glasses. (a) Theoretical and measured spectral absorptance in region of 0.3-25 μm. Here, it is noted that the liquid water has no absorption in the UV spectrum according to the calculation. The measured absorption of the hydrogel is caused by the polymer networks in the hydrogel. The solid and dashed line represent the measured and calculated results, respectively. (b-c) Measured spectral transmittance (b) and reflectance (c) in the range of 0.3-2.5 μm. Figure S4. Schematic of the calculations of the transmittance and reflectance of the hydrogelglass. Figure S5. Light management of solar cells with hydrogel-glass with different thick hydrogel layers. (a) Photograph of the solar cells. The ccale bar is 4 cm. (b-c) Photovoltaic efficiencies (b) and working temperatures (c) of the solar cells.(d) Measured spectral reflectance of the solar cells in 0.3-2.5 μm. Figure S6. Experiment setup to test the radiative cooling performance of hydrogel-glass. (a) Schematic of the setup. (b) Photograph of the experiment setup. Figure S7. Performance demonstration of the hydrogel-glass in a house model. (a) Photograph on experiment setup. (b) Temperatures of the hydrogel-glass, normal glass and environment. Table S1 Optical and thermal properties of three windows used in the simulations. Ta [file 12200_2022_33_MOESM1_ESM.docx]

**Supporting information for**

**Broad Light Management in Hydrogel Glass for Energy Efficient Windows**

By Jia Fu *et al*.

**Additional notes**

**Note 1. Theoretical calculations of the reflectance, transmittance and absorptance of the hydrogel-glass.**

Since the wavelength of photon is much smaller than the thickness of hydrogel, the interference effect between the two boundaries of hydrogel can be ignored. Hence, Ray Tracing method was used to calculate the spectral reflectance and transmittance of the hydrogel-glass, as shown in Figure S4. In order to simplify the calculations, the reflection at the hydrogel-glass interface was also ignored, due to the close refraction index between hydrogel (1.33) and glass (1.50). Here, the thickness of hydrogel layer is assumed to be *d.* The surface reflectance *ρ*(λ) between air and hydrogel (glass) can be calculated by

$\rho(\lambda)=\frac{{(n-1)}^{2}+k^{2}}{{(n+1)}^{2}+k^{2}}$ (S1)

The internal transmittance *τ*(λ) can be calculated by

$\tau(\lambda)=exp(-\frac{4\pi kd}{\lambda}$) (S2)

Hence, the spectral reflectance *R*_λ_ and transmittance of the hydrogel-glass can be obtained as

$R_{\lambda}={\rho(\lambda)}_{w}+\frac{{\rho\left( \lambda\right)}_{G}\left[ 1-{\rho(\lambda)}_{w} \right]^{2}{\tau(\lambda)}^{2}}{1-{\rho\left( \lambda\right)}_{G}{\rho(\lambda)}_{w}{\tau(\lambda)}^{2}}$ (S3)

$T_{\lambda}=\frac{\left[ 1-{\rho(\lambda)}_{w} \right]\left[ 1-{\rho\left( \lambda\right)}_{G} \right]\tau}{1-{\rho\left( \lambda\right)}_{G}{\rho(\lambda)}_{w}\tau^{2}}$ (S4)

${\rho(\lambda)}_{w}$ is the surface reflectance between air and hydrogel. ${\rho\left( \lambda\right)}_{G}$ is the surface reflectance between glass and air.

The spectral absorptance can be described as

$A(\lambda)=1-R(\lambda)-T(\lambda)$ (S5)

The average visible transmittance$\bar{T}$ is calculated as

$\bar{T}(0.38-0.76\mu m)=\frac{\int_{0.38}^{0.76} d\lambda\cdot\tau(\lambda)\cdot I_{AM1.5}(\lambda)}{\int_{0.38}^{0.76} d\lambda\cdot I_{AM1.5}(\lambda)}$ (S6)

$I_{AM1.5}(\lambda)$ is the solar radiation spectrum of the AM1.5.

The average thermal emittance $\bar{\varepsilon}$ in the transparent atmosphere window spectrum can be evaluated as

$\bar{\varepsilon}=\frac{\int_{8\mu m}^{14\mu m} d\lambda\cdot\varepsilon(\lambda)\cdot I_{BB}(T, \lambda)}{\int_{8\mu m}^{14\mu m} d\lambda\cdot I_{BB}(T, \lambda)}$ (S7)

$I_{BB}(T, \lambda)$ is the spectral radiance of the blackbody at temperature *T*. *T* is taken as 25 °C.

Based on the optical constants (refractive index *n* and extinction coefficient *κ*) of silica and water in reference 1 and 2 ^1, 2^, we can obtain spectral absorptance in 0.3-25 μm of hydrogel-glass with different thicknesses in Figure S3, as well as the spectral transmittance of the hydrogel-glass in visible spectrum as Figure 2b.

**Note 2. Thermal conductivity of the hydrogel-glass.**

The thermal conductivity (*λ*_1_) of the hydrogel layer was measured by transient hot-wire method (TC3000E, XIATECH). While the thermal conductivity (*λ*_2_ = 0.9 W/(m·k)) of the glass was obtained from the EngergyPlus Software (version 9.5.1). Then, the effective thermal conductivity of the hydrogel-glass with different thickness of hydrogel layer can be calculated by

$\lambda=\frac{D_{1}+D_{2}}{\frac{D_{1}}{\lambda_{1}}+\frac{D_{2}}{\lambda_{2}}}$ (S8)

*λ*_1_ and *λ*_2_ are the thicknesses of hydrogel and glass. *D*_1_ was taken as 4 mm and *D*_2_ was taken as 6 mm. *λ*_1_ is measured to be 0.47 W/(m·k). *λ* is 0.66 W/(m·k) according to Equation of S8 (Table S1). Here, the contact resistance between the glass and the hydrogel is small and could be neglected due to the chemical bonding between them.

**Note 3. Photon penetration depth of water and glass.**

The absorption of a beam of radiation at it propagates through an absorbing medium is described by the Beer-Lambert law:

$T(\lambda)=e^{-\alpha_{\lambda}d}$ (S9)

$T(\lambda)$ is the spectral transmittance and *d* is the distance. $\alpha_{\lambda}$ is the spectral absorption coefficient, which can be calculated as

$\alpha_{\lambda}=\frac{4\pi k}{\lambda}$ (S10)

*κ* is the extinction coefficient of the materials. Thus, the photon penetration depth $\delta(\lambda)$ is defined as 1/$\alpha_{\lambda}$, and can be calculated by

$\delta(\lambda)=\frac{\lambda}{4\pi k}$ (S11)

For calculating the photon penetration depth of glass and water, the extinction coefficient *κ* is obtained from the reference 1 and 2, respectively ^1, 2^.

**Additional Figures**


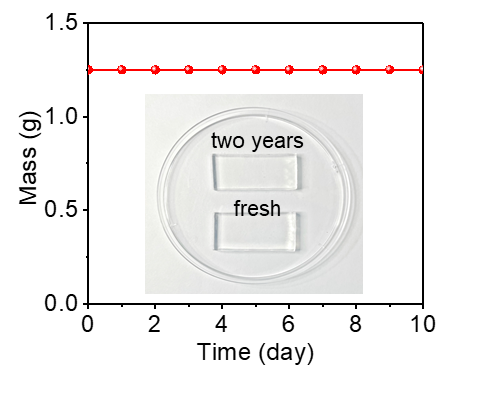


**Figure S1** Mass variation of the hydrogel exposed in ambient environment. The size of the samples is 1cm × 2 cm × 2 mm. The ambient temperature is about 26 °C, and the relative humidity is about 50%. Inset shows the hydrogel after storage in environment for two years and the newly prepared hydrogel.

**
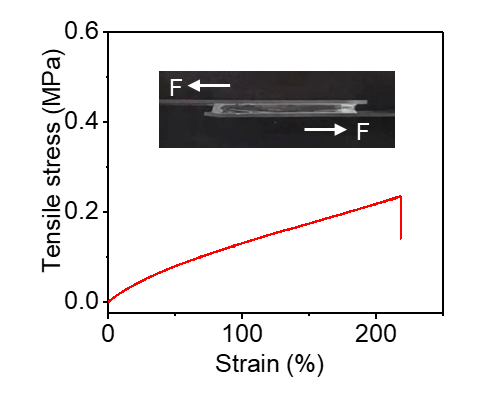
**

**Figure S2** Stress-strain curve of the hydrogel sandwich between two glass plates. Inset is the photograph during the test.


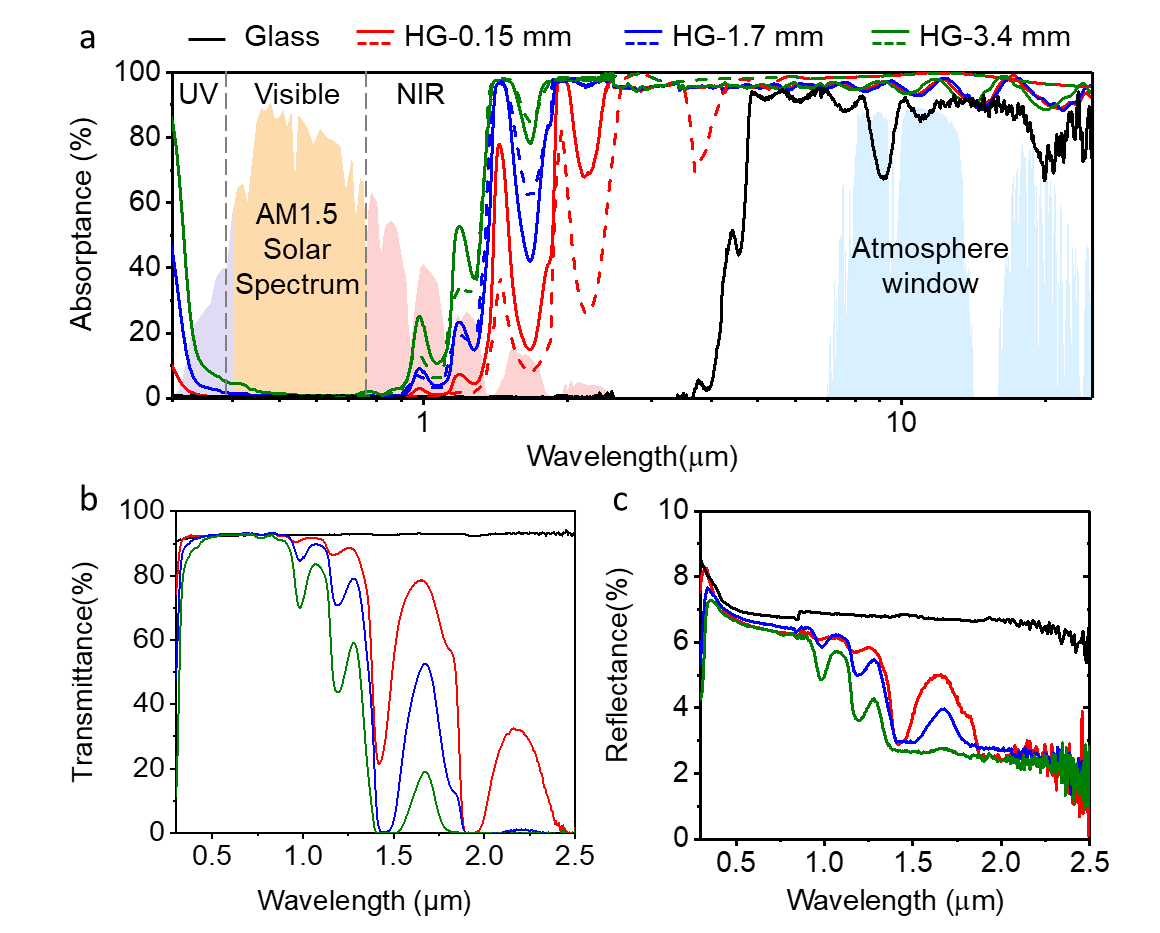


**Figure S3** Optical spectra of the glass and hydrogel-glasses. (a) Theoretical and measured spectral absorptance in region of 0.3-25 μm. Here, it is noted that the liquid water has no absorption in the UV spectrum according to the calculation. The measured absorption of the hydrogel is caused by the polymer networks in the hydrogel. The solid and dashed line represent the measured and calculated results, respectively. (b-c) Measured spectral transmittance (b) and reflectance (c) in the range of 0.3-2.5 μm.


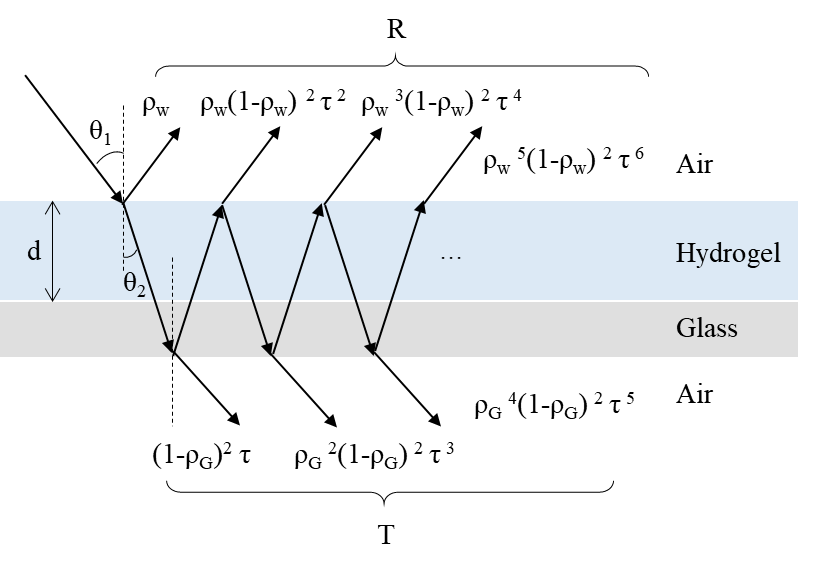


**Figure S4** Schematic of the calculations of the transmittance and reflectance of the hydrogel-glass.


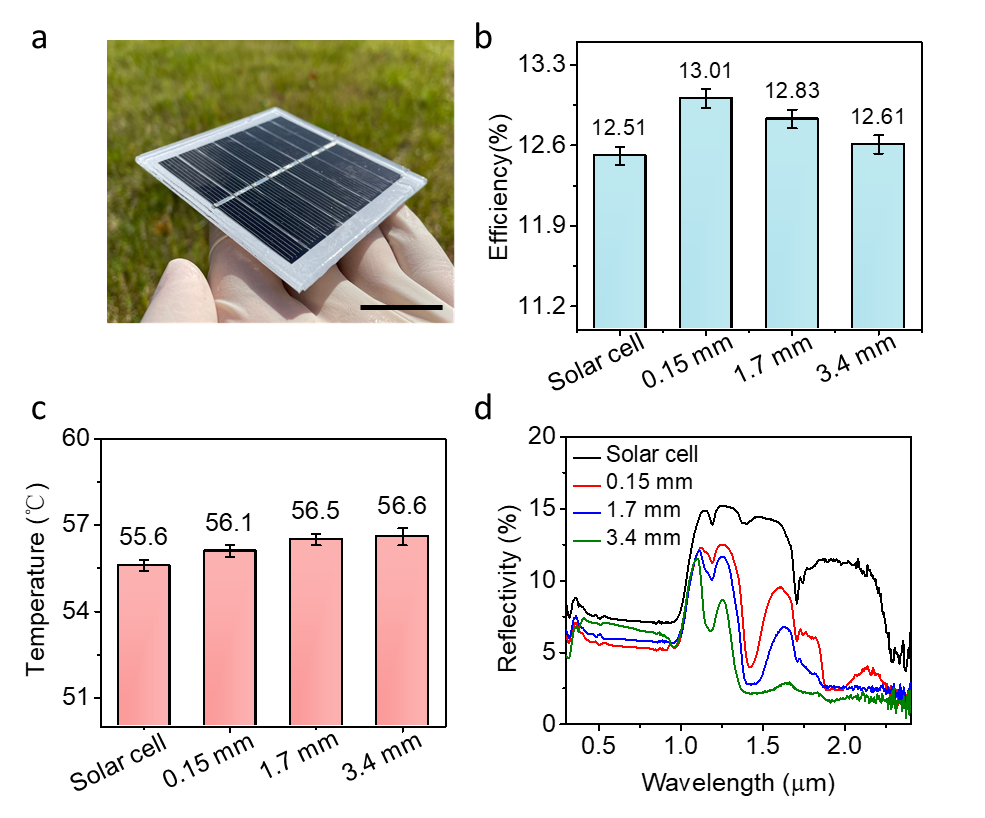


**Figure S5** Light management of solar cells with hydrogel-glass with different thick hydrogel layers. (a) Photograph of the solar cells. The ccale bar is 4 cm. (b-c) Photovoltaic efficiencies (b) and working temperatures (c) of the solar cells. (d) Measured spectral reflectance of the solar cells in 0.3-2.5 μm.


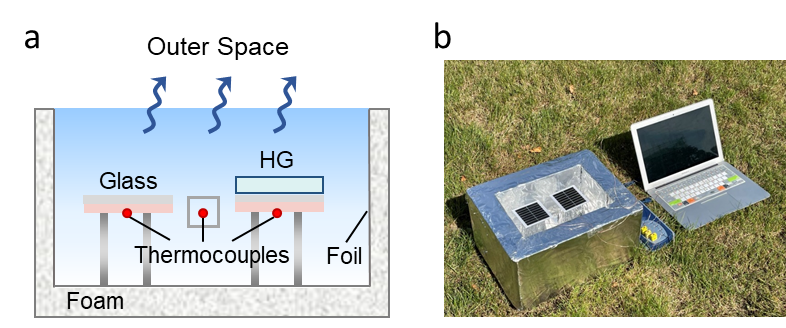


**Figure S6** Experiment setup to test the radiative cooling performance of hydrogel-glass. (a) Schematic of the setup. (b) Photograph of the experiment setup.


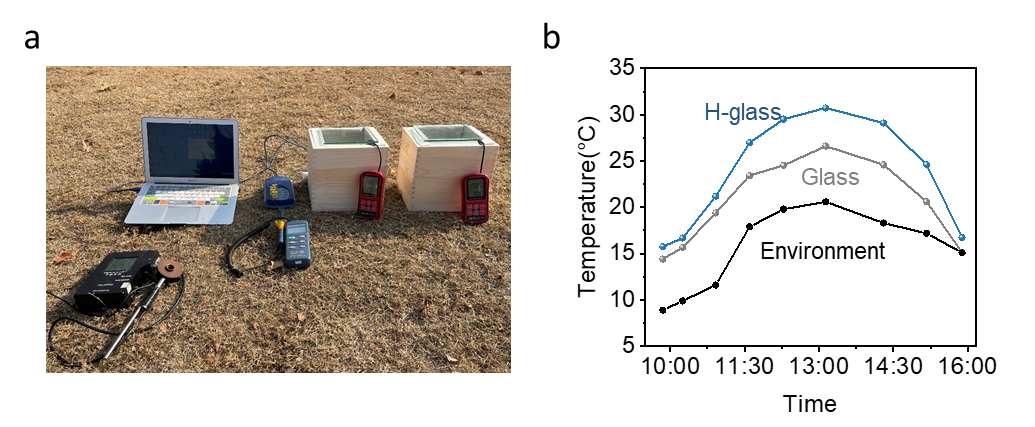


**Figure S7** Performance demonstration of the hydrogel-glass in a house model. (a) Photograph on experiment setup. (b) Temperatures of the hydrogel-glass, normal glass and environment.

**Table S1** Optical and thermal properties of three windows used in the simulations.

| **Parameters** | **Glass** | **TNS Glass** | **Hydrogel Glass** |
| --- | --- | --- | --- |
| Thickness(m) | 0.006 | 0.006 | 0.01 |
| Solar Transmittance at Normal Incidence | 0.815 | 0.43 | 0.65 |
| Visible Transmittance at Normal Incidence | 0.88 | 0.68 | 0.89 |
| Outside Infrared Hemisphercial Emissivity | 0.84 | 0.64 | 0.96 |
| Inside Infrared Hemisphercial Emissivity | 0.84 | 0.84 | 0.84 |
| Thermal Conductivity  (W/(m·k)) | 0.9 | 0.9 | 0.66 |

**Table S2** Climatic classification and geographical location of eight cities.

| **City** | **Climate** | **Location** |
| --- | --- | --- |
| Moscow | Temperate Continental Climate | 55.75°N, 37.63°E |
| Lhasa | Plateau Mountain Climate | 29.67°N, 91.13°E |
| Beijing | Monsoon Climate of Medium Latitudes | 39.8°N, 116.47°E |
| London | Temperate Marine Climate | 51.15°N, -0.18°E |
| Wuhan | Subtropical Monsoon Climate | 30.62°N, 114.13°E |
| Haikou | Tropical Maritime Monsoon Climate | 20.03°N, 110.35°E |
| Los. Angeles | Etesian Climate | 34.03°N, -118.22°E |
| Aswan | Tropical Desert Climate | 23.97°N, 32.78°E |

**Table S3** Annual electricity consumption and energy saving potential in the eight cities.

| **City** | **Normal glass** | | **H-glass** | | **Energy saving**  (MJ/m^2^) |
| --- | --- | --- | --- | --- | --- |
|  | Lighting  (MJ/m^2^) | Cooling  (MJ/m^2^) | Lighting  (MJ/m^2^) | Cooling  (MJ/m^2^) |  |
| Lhasa | 44.56 | 17.94 | 44.52 | 15.61 | 2.37 |
| London | 50.97 | 22.4 | 50.93 | 19.77 | 2.67 |
| Moscow | 50.77 | 23.55 | 50.72 | 20.81 | 2.79 |
| Beijing | 45.31 | 64.91 | 45.29 | 60.24 | 4.69 |
| Wuhan | 46.65 | 89.57 | 46.62 | 84.04 | 5.56 |
| Los. Angeles | 48.52 | 77.57 | 48.5 | 69.76 | 7.83 |
| Haikou | 46.44 | 148.99 | 46.40 | 139.82 | 9.21 |
| Aswan | 49.93 | 149.36 | 49.90 | 138.94 | 10.45 |

**Additional References**

[1] Palik ED. Handbook of optical constants of solids, vol. 1. Academic press, 1998.

[2] Palik ED. Handbook of optical constants of solids, vol. 2. Academic press, 1998.
